# Prevalence and predictors of water-borne diseases among elderly people in India: evidence from Longitudinal Ageing Study in India, 2017–18

**DOI:** 10.1186/s12889-022-13376-6

**Published:** 2022-05-17

**Authors:** Pradeep Kumar, Shobhit Srivastava, Adrita Banerjee, Snigdha Banerjee

**Affiliations:** 1 Indian Health Action Trust, Lucknow, India; 2 Mamta Health Institute for Mother and Child, Delhi, India; 3 International Institute for Population Sciences, Mumbai, India

**Keywords:** Water-borne diseases, Older adults, Rural–urban divide, India

## Abstract

**Background:**

India suffers from a high burden of diarrhoea and other water-borne diseases due to unsafe water, inadequate sanitation and poor hygiene practices among human population. With age the immune system becomes complex and antibody alone does not determine susceptibility to diseases which increases the chances of waterborne disease among elderly population. Therefore the study examines the prevalence and predictors of water-borne diseases among elderly in India.

**Method:**

Data for this study was collected from the Longitudinal Ageing Study in India (LASI), 2017–18. Descriptive statistics along with bivariate analysis was used in the present study to reveal the initial results. Proportion test was applied to check the significance level of prevalence of water borne diseases between urban and rural place of residence. Additionally, binary logistic regression analysis was used to estimate the association between the outcome variable (water borne diseases) and the explanatory variables.

**Results:**

The study finds the prevalence of water borne disease among the elderly is more in the rural (22.5%) areas compared to the urban counterparts (12.2%) due to the use of unimproved water sources. The percentage of population aged 60 years and above with waterborne disease is more in the central Indian states like Chhattisgarh and Madhya Pradesh followed by the North Indian states. Sex of the participate, educational status, work status, BMI, place of residence, type of toilet facility and water source are important determinants of water borne disease among elderly in India.

**Conclusion:**

Elderly people living in the rural areas are more prone to waterborne diseases. The study also finds state wise variation in prevalence of waterborne diseases. The elderly people might not be aware of the hygiene practices which further adhere to the disease risk. Therefore, there is a need to create awareness on basic hygiene among this population for preventing such bacterial diseases.

**Supplementary Information:**

The online version contains supplementary material available at 10.1186/s12889-022-13376-6.

## Introduction

The Sustainable Development Goal, 2017 aimed to ensure availability and sustainable management of water and sanitation for all by 2030 [1]. However globally 780 million people live without access to safe water and approximately 2.5 billion people in the developing world lived without access to adequate sanitation [2, 3]. Polluted water and poor sanitation practices expose individuals to health risks. Emerging water-borne pathogens constitute a significant health hazard in both developed and developing nations [4] as they can spread rapidly and affect large sections of the population. Water-borne diseases are transmitted through contaminated drinking water with pathogen microorganisms such as protozoa, virus, bacteria, and intestinal parasites. According to the projection of Global Burden Disease report, the burden of water borne disease was the second highest reason for mortality in 1990 however, it was lower down in ninth most important reason for mortality in 2020 [5]. Around 829,000 people are estimated to die each year from diarrheal diseases majorly cholera, dysentery and typhoid fever due to unsafe drinking water and unhygienic sanitation practice [6]. Further, the WHO (2015) reported that about 6.3 per cent of deaths occur due to unsafe water, inadequate sanitation, and poor hygiene. Adequate, safe, and accessible water supplies as well as satisfactory sanitation are most required to have secure health status [7]. According to WHO (2015), nearly 4 percent of the global disease burden could be prevented by improving water supply, sanitation, and hygiene [8].

It is estimated that around 37.7 million Indians are affected by waterborne diseases annually; 1.5 million children are estimated to die of diarrhoea alone and 73 million working days are lost due to waterborne disease each year [9]. Water-borne diseases pose a high disease burden and significantly impact on country’s economic growth [10]. These diseases erupt every year during summer and rainy seasons as a result of improper management of water supply especially of drinking water and sanitation [11, 12].

Poor urban governance, rapidly growing economies, highly dense population, poor housing and sanitation in slum areas of cities create environments rife for waterborne diseases [13]. One of the study in slum areas of Mumbai revealed that at least 30 per cent of all morbidity are due to water-related infections [14]. In rural areas, there are no proper water supply and sewerage systems. In the villages, water contamination can be attributed to infiltration, leaching, and surface run-off through pastures, lacking and leakage of sewerage disposal systems. Studies based on rural India revealed that lacking in knowledge, attitudes and practices (KAP) with regard to water handling, sanitation and defecation practices are common causes of waterborne diseases [15, 16]. Water pollution, open defecation and poor hygiene practices are the main hindrances to achieving good health. Therefore, safe and readily available water is essential for public health whether used for drinking, domestic use, food production or recreational purposes. Adequate access to safe water, improving quality of water source, treating and storing household water and encouraging hygiene practices can prevent waterborne diseases. As the global population is increasing rapidly over time, water availability will lower down steadily [8]. Individuals with low immunity are more susceptible to water-borne diarrheal diseases, especially children andelderly, with the low immune system are most susceptible to pathogen-related water-borne diseases [17]. According to U.S. Environmental Protection Agency, elderly along with children and pregnant women, were recognized as the sensitive sub-populations for water-borne diseases [18].

India is currently in the third stage of demographic transition and with 8% of geriatric population India could well be called an ageing nation [19]. The elderly population of India is expected to increase three fold by 2050 [20]. Among the elderly, infections are often more severe due to the presence of multiple underlying medical conditions, low immune system, and frequent use of drugs [21]. People in India mostly are unaware of safe and hygienic practices and this is prevalent across all age groups. This in turn increases the risk of communicable diseases. Thus, the resources and policy attention should be focused on strengthening primary health care systems that address communicable diseases and reduce the underlying risk factors. The rising number of elderly with various health problems creates a pressure on the existing public health system in India. In order to focus on strengthening the health care system to serve the elderly population there is need to study the prevalence of various disease risk among the elderly population. The major objective of the study is to examine the prevalence and predictors of water borne diseases among elderly. The study aims to bridge the research gap as less attention has been paid on the water borne diseases among the elderly population. The results of the study would further help in embarking knowledge, attitude and practices related to water handling, sanitation and defecation practices among the elderly which might reduce to some extent the load of communicable disease risk among elderly.

## Materials and methods

### Data

#### Study settings and population

Cross-sectional data for this study was used from the Longitudinal Ageing Study in India (LASI), nationally representative survey conducted in the year 2017–18 and covered 72,000 elderly age 45 and above across all states and union territories of India [22].

### Study design

Cross-sectional survey.

### Sample size calculation and sampling procedure

LASI is a full-scale national survey of scientific investigation of the health, economic, and social determinants and consequences of population aging in India. The main objective of the LASI survey was to study the health status and the social and economic well-being of elderly in India. The survey adopted a multistage stratified area probability cluster sampling design to arrive at the eventual units of observation: elderly age 45 and above and their spouses irrespective of age.

Within each state, LASI Wave 1 adopted three-stage sampling design in rural areas and four-stage sampling design in urban areas. In each state/UTs, the first stage involved selection of Primary Sampling Units (PSUs), that is, sub-districts (Tehsils/Talukas), and the second stage involved the selection of villages in rural areas and wards in urban areas in the selected PSUs. In rural areas, households were selected from selected villages in the third stage. However, sampling in urban areas involved an additional stage. Specifically, in the third stage, one Census Enumeration Block (CEB) was randomly selected in each in urban area. In the fourth stage, households were selected from this CEB.

The present study is conducted on the eligible participant’s age 60 years and above. The total sample size for the present study is 31464 (for rural-20725 and urban-10739) elders aged 60 years and above [22].

### Study variables

#### Outcome variable

The outcome variable (water borne diseases) was binary in nature i.e. water borne diseases coded as no and yes. The variable was generated using the question “has any health professional diagnosed you with diarrhoea/gastroenteritis or typhoid or jaundice/hepatitis in last two years [23].

#### Explanatory variables

The control variables were selected after doing extensive literature review. The variables selected are as follows:

1. Age was recoded as 60–69, 70–79 and 80 + years.
2. Sex was recoded as male and female.
3. Education was recoded as no education/primary not completed, primary completed, secondary completed and higher and above.
4. Marital status was recoded as currently married, widowed and others. Others included separated/never married/divorced.
5. Working status was coded as currently working, retire and never worked.
6. Body mass index was recoded as underweight, normal and overweight/obese. The participants having a body mass index (BMI) of 25 and above were categorized as obese/overweight whereas participant who had BMI as 18.4 and less were coded as underweight [24]. BMI is calculated by dividing an individual’s weight (in kilograms) by the square of their height (in metres).
7. Type of toilet facility was recoded as unimproved and improved [25]. Improved toilet facility includes pour-flush latrines, ventilated improved pit latrines, and pit latrines with a slab/covered pit. Unimproved toilet facility includes Shared facilities of any type, no facilities (bush or field); flush or pour-flush to elsewhere (that is, not to piped sewer system, septic tank or pit latrine); pit latrines without slab / open pits, bucket systems; hanging toilet or hanging latrine.
8. Source of drinking water was recoded as unimproved and improved [25]. Improved source of drinking water includes piped water, public tap/standpipe, tube well or bore well, dug well, spring water and rain water. Unimproved water sources include tanker, cart with small tank, bottled water/pouch water, surface water and other sources of water.
9. Type of house was recoded as pucca, semi pucca and kutcha.
10. The monthly per capita expenditure (MPCE) was assessed using household consumption data. Sets of 11 and 29 questions on the expenditures on food and non-food items, respectively, were used to canvas the sample households. Food expenditure was collected based on a reference period of seven days, and non-food expenditure was collected based on reference periods of 30 days and 365 days. Food and non-food expenditures have been standardized to the 30-day reference period. The monthly per capita consumption expenditure (MPCE) is computed and used as the summary measure of consumption. The variable was then divided into five quintiles i.e., from poorest to richest [22].
11. Religion was recoded as Hindu, Muslim, Christian and Others.
12. Caste was recoded as Scheduled Tribe, Scheduled Caste, Other Backward Class, and others. The Scheduled Caste include “untouchables”; a group of the population that is socially segregated and financially/economically by their low status as per Hindu caste hierarchy. The Scheduled Castes (SCs) and Scheduled Tribes (STs) are among the most disadvantaged socio-economic groups in India. The OBC is the group of people who were identified as “educationally, economically and socially backward”. The OBC’s are considered low in the traditional caste hierarchy but are not considered untouchables. The “other” caste category is identified as having higher social status [26].
13. Place of residence was recoded as rural and urban area.
14. Region was recoded as North, Central, East, Northeast, West, and South.

### Statistical analysis

Univariate along with bivariate analysis was used in present study to reveal the initial results. Proportion test [27] was applied to check the significance level of prevalence of water borne diseases between urban and rural place of residence. Additionally, binary logistic regression analysis [28] was used to estimate the association between the outcome variable (water borne diseases) and other explanatory variables.

The binary logistic regression model is usually put into a more compact form as follows:

$$\mathrm{Logit }\left[\mathrm{P}\left(\mathrm{Y}=1\right)\right]={\beta }_{0}+\beta *X+ \epsilon$$

The parameter ${\beta }_{0}$ estimates the log odds of water borne diseases for the reference group, while $\beta$ estimates the maximum likelihood, the differential log odds of water borne diseases associated with a set of predictors X, as compared to the reference group, and $\epsilon$ represents the residual in the model. The variance inflation factor (VIF) ( Additional file -Table-A1) was used to check for the presence of multicollinearity and the test confirmed that there was no evidence of multicollinearity [29]. STATA 14 was used for the analysis purpose.

## Results

### Socio-demographic and economic profile of elderly in India

Table 1 presents the socio-demographic and economic profile of the study participants. A similar proportion of elderly lived in rural and urban areas irrespective of age group. Only three per cent of elderly in rural areas had higher education and this percentage was five times in urban areas. In rural areas, about one-third of elderly were working whereas one-fifth of elderly in urban areas were working. Nearly one-third of older adults in rural and one in every ten older elderly in urban areas were underweight. Only one-third of elderly in rural areas were used improved toilet facility and eight in every 10 elderly in urban areas were used improved toilet facility. In rural areas, three fifth of elderly used improved source of drinking water whereas nine in every ten elderly from urban areas used improved source of drinking water. About four in every ten elderly in rural areas lived in pucca house and this proportion was almost double in urban areas.

**Table 1:** Socio-demographic and economic profile of elderly in India

Background characteristics	Rural		Urban	
	**Sample**	**%**	**Sample**	**%**
**Age (in years)**				
60–69	12,139	58.6	6268	58.4
70–79	6169	29.8	3354	31.2
80 +	2417	11.7	1117	10.4
**Sex**				
Male	10,045	48.5	4835	45.0
Female	10,680	51.5	5904	55.0
**Education**				
No education/primary not completed	15,984	77.1	4937	46.0
Primary completed	2069	10.0	1511	14.1
Secondary completed	1988	9.6	2598	24.2
Higher and above	682	3.3	1693	15.8
**Marital status**				
Currently married	13,017	62.8	6315	58.8
Widowed	7280	35.1	4162	38.8
Others	427	2.1	262	2.4
**Working status**				
Working	7341	35.4	2106	19.6
Retired	8774	42.3	4719	43.9
Not working	4610	22.2	3913	36.4
**Body Mass Index**				
Underweight	6062	32.4	1142	12.2
Normal	9742	52.1	4561	48.7
Overweight/obese	2884	15.4	3658	39.1
**Type of toilet facility**				
Unimproved	13,455	64.9	1984	18.5
Improved	7270	35.1	8755	81.5
**Source of drinking water**				
Unimproved	8035	38.8	1319	12.3
Improved	12,690	61.2	9420	87.7
**Type of house**				
Pucca	8512	41.8	8281	80.0
Semi pucca	7064	34.7	1646	15.9
Kutcha	4794	23.5	428	4.1
**MPCE quintile**				
Poorest	4446	21.5	2396	22.3
Poorer	4608	22.2	2197	20.5
Middle	4375	21.1	2207	20.6
Richer	3932	19.0	2117	19.7
Richest	3364	16.2	1822	17.0
**Religion**				
Hindu	17,309	83.5	8497	79.1
Muslim	2021	9.8	1604	14.9
Christian	623	3.0	269	2.5
Others	772	3.7	369	3.4
**Caste**				
Scheduled Caste	4572	22.1	1220	11.4
Scheduled Tribe	2125	10.3	325	3.0
Other Backward Class	9213	44.5	5056	47.1
Others	4815	23.2	4139	38.5
**Region**				
North	2655	12.8	1293	12.0
Central	4920	23.7	1533	14.3
East	5678	27.4	1573	14.7
Northeast	691	3.3	226	2.1
West	2898	14.0	2662	24.8
South	3883	18.7	3451	32.1

Weighted estimates are presented in the table

**Fig. 1:**
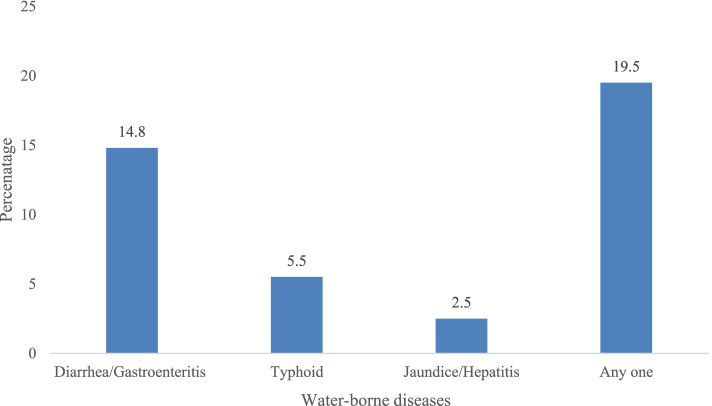
Prevalence of diarrhoea/gastroenteritis or typhoid or jaundice/hepatitis among elderly in India, 2017–18

Figure 1 shows the prevalence of diarrhoea/gastroenteritis or typhoid or jaundice/hepatitis. It was found that 14.8% (14.4–15.2) of elderly suffered from diarrhoea/gastroenteritis and 5.5% (5.2–5.7) suffered from typhoid and 2.5% (2.3–2.7) suffered from jaundice/hepatitis. The prevalence of water borne diseases among elderly was 19.5% (19.0–19.8).

### Prevalence of water borne diseases among elderly in India

Table 2 shows that there was a significant rural–urban difference in the prevalence of water borne diseases in India (difference: 10.2 percentage point). The prevalence of water borne disease among elderly in rural areas was 22.5% whereas in urban areas the prevalence was 12.2%. The rural–urban differences was highest among elderly who used unimproved toilet facility (difference: 17.1 percentage point), had 80 + years of age (difference: 14.4 percentage point), who belonged to other backward class (difference: 12.4 percentage point), richer elderly (difference: 12.3 percentage point), and those were not working (difference: 12.1 percentage point). Moreover, the prevalence of water borne diseases was higher among underweight elderly, and those who lived in kutcha houses irrespective to their place of residence.

**Table 2:** Percentage of elderly suffering from water borne diseases by their background characteristics in India

Background characteristics	Rural	Urban	Difference	*p*-value
	**%**	**%**	**%**	
**Age (in years)**				
60–69	22.4	12.2	10.2	<0.001
70–79	21.9	13.0	8.9	<0.001
80 +	24.2	9.8	14.4	<0.001
**Sex**				
Male	21.6	12.2	9.4	<0.001
Female	23.3	12.3	11.1	<0.001
**Education**				
No education/primary not completed	23.4	14.7	8.7	<0.001
Primary completed	20.4	12.2	8.2	<0.001
Secondary completed	19.5	9.2	10.3	<0.001
Higher and above	14.9	9.6	5.3	<0.001
**Marital status**				
Currently married	22.3	12.0	10.3	<0.001
Widowed	23.1	12.7	10.4	<0.001
Others	18.5	9.8	8.7	<0.001
**Working status**				
Working	22.4	13.4	9.1	<0.001
Retired	21.9	12.3	9.6	<0.001
Not working	23.6	11.5	12.1	<0.001
**Body Mass Index**				
Underweight	25.9	17.7	8.2	<0.001
Normal	21.9	14.1	7.8	<0.001
Overweight/obese	20.5	9.0	11.5	<0.001
**Type of toilet facility**				<0.001
Unimproved	26.6	9.4	17.1	<0.001
Improved	19.9	12.6	7.3	<0.001
**Source of drinking water**				
Unimproved	17.8	11.1	6.8	0.024
Improved	22.8	12.4	10.3	<0.001
**Type of house**				
Pucca	20.7	12.3	8.4	<0.001
Semi pucca	22.0	14.6	7.4	<0.001
Kutcha	26.5	15.6	10.8	<0.001
**MPCE quintile**				
Poorest	23.6	14.8	8.8	<0.001
Poorer	24.3	14.6	9.7	<0.001
Middle	20.8	11.7	9.1	<0.001
Richer	22.3	10.0	12.3	<0.001
Richest	20.7	9.2	11.5	<0.001
**Religion**				
Hindu	22.8	11.8	11.0	<0.001
Muslim	24.0	14.6	9.4	<0.001
Christian	13.4	7.3	6.2	0.987
Others	19.5	16.1	3.4	0.024
**Caste**				
Scheduled Caste	23.6	14.2	9.4	<0.001
Scheduled Tribe	23.0	20.0	3.0	<0.001
Other Backward Class	22.8	10.3	12.4	<0.001
Others	20.6	13.3	7.3	<0.001
**Region**				
North	28.7	21.4	7.3	<0.001
Central	34.7	26.7	8.0	<0.001
East	22.3	16.3	6.1	<0.001
Northeast	13.5	12.5	1.0	<0.001
West	15.2	8.3	6.9	<0.001
South	9.9	3.5	6.4	<0.001
**Total**	22.5	12.2	10.2	<0.001

Weighted estimates are presented in the table

**Fig. 2:**
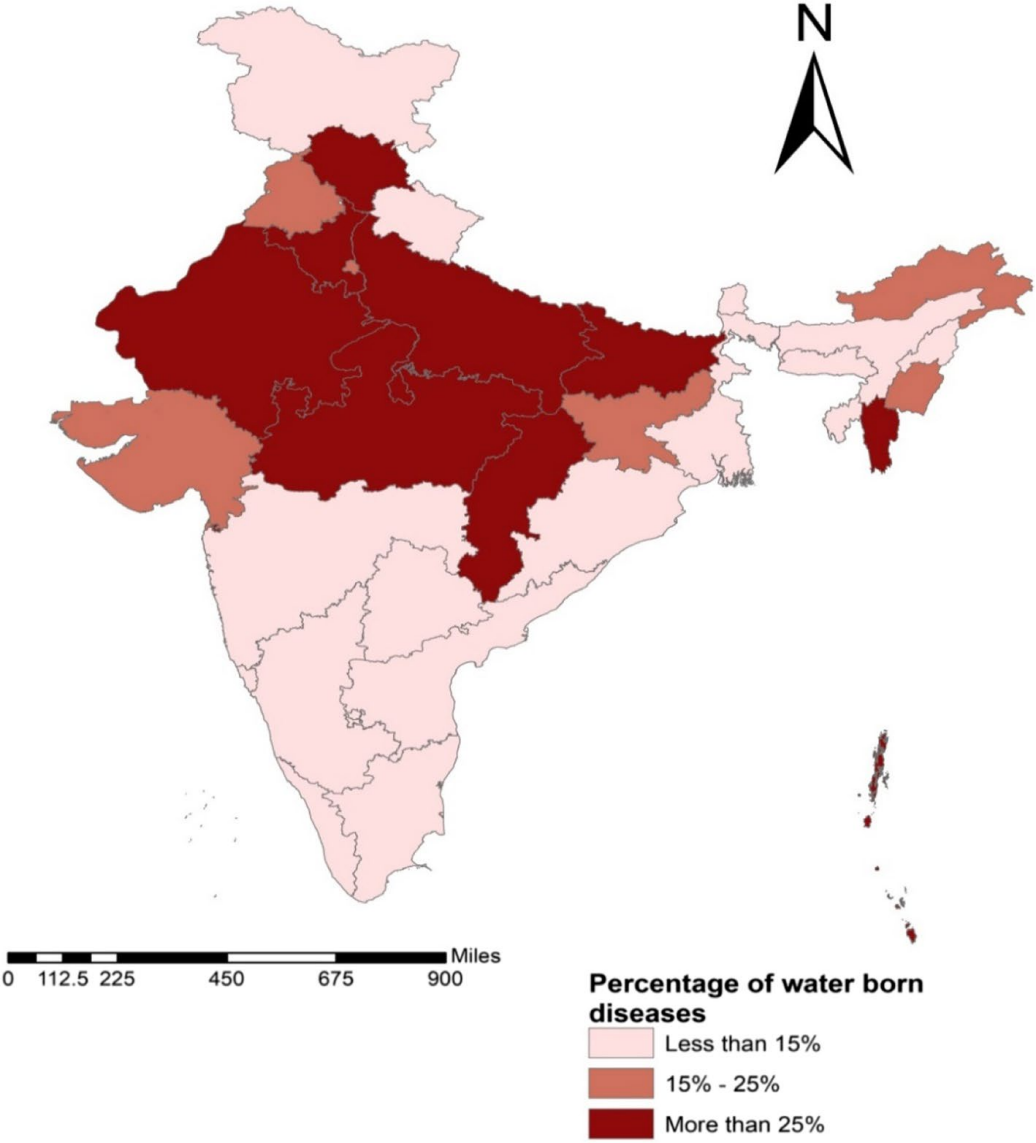
shows state-wise prevalence of water borne diseases among elderly in India

Figure 2 shows state-wise prevalence of water borne diseases among elderly in India. The prevalence of water borne diseases was highest in Chhattisgarh (36.9 per cent), followed by Mizoram (35 per cent), Haryana (34.6 per cent), and Bihar (34 per cent). However, this prevalence was lowest in Kerala (3.5 per cent), followed by Goa (6.2 per cent), and Tamil Nadu (6.8 per cent).

#### State-wise prevalence of water borne diseases in rural and urban areas in India

Table 3 presents the state-wise prevalence of water borne disease stratified by place of residence in India. In rural areas, the prevalence of water borne diseases was highest in Chhattisgarh (38.5 per cent) followed by Madhya Pradesh (36 per cent), Haryana (35.2 per cent), and Rajasthan (34.9 per cent) while for urban areas, water borne diseases was more prevalent in Bihar (36.3 per cent), followed by Mizoram (36.1 per cent), Himachal Pradesh (32.9 per cent), and Haryana (32.4 per cent) [Additional file Table A2].

**Table 3:** Percentage of elderly suffered from water borne diseases in states of India (rural and urban)

States	Rural (%)	Urban (%)	*p*-value
Jammu & Kashmir	10.4	6.6	< 0.001
Himachal Pradesh	28.7	32.9	< 0.001
Punjab	22.0	23.9	0.108
Chandigarh	0.0	11.5	< 0.001
Uttarakhand	11.6	14.2	< 0.001
Haryana	35.2	32.4	< 0.001
Delhi	0.0	17.5	< 0.001
Rajasthan	34.9	25.3	< 0.001
Uttar Pradesh	33.6	26.7	< 0.001
Bihar	33.7	36.3	< 0.001
Arunachal Pradesh	24.6	9.9	< 0.001
Nagaland	1.2	2.8	< 0.001
Manipur	19.7	24.1	< 0.001
Mizoram	34.5	36.1	< 0.001
Tripura	13.5	8.6	< 0.001
Meghalaya	9.5	5.5	< 0.001
Assam	12.6	7.2	< 0.001
West Bengal	14.9	10.1	< 0.001
Jharkhand	18.1	12.5	< 0.001
Odisha	11.1	8.1	< 0.001
Chhattisgarh	38.5	30.4	< 0.001
Madhya Pradesh	36.0	26.1	< 0.001
Gujarat	22.3	14.5	< 0.001
Daman & Diu	20.1	12.8	< 0.001
Dadra & Nagar Haveli	44.9	42.0	< 0.001
Maharashtra	12.8	4.9	< 0.001
Andhra Pradesh	9.4	3.3	< 0.001
Karnataka	14.5	1.9	< 0.001
Goa	8.6	4.1	< 0.001
Lakshadweep	7.3	1.5	< 0.001
Kerala	3.9	3.5	0.665
Tamil Nadu	7.7	5.0	0.567
Puducherry	8.9	1.7	< 0.001
Andaman & Nicobar Island	26.0	18.2	< 0.001
Telangana	8.8	8.3	0.287
**India**	**22.5**	**12.2**	< 0.001

### Estimates from logistic regression analysis for older adults who suffered from water borne diseases in India

Table 4 shows the adjusted odds ratio for elderly  who suffered from water borne disease in India. It was revealed that the odds of water borne diseases was high in rural areas in reference to urban areas [AOR: 1.21; *p* < 0.05]. The likelihood of water borne diseases was significantly more among elderly female than male counterparts [AOR: 1.19; *p* < 0.05]. Moreover, the odds of water borne diseases were decreased with increase the level of education among elderly. The risk of water borne diseases was 12 per cent more among underweight elderly compared to overweight/obese elderly [AOR: 1.12; *p* < 0.05]. Similarly, elderly who used unimproved toilet facility [AOR: 1.22; *p* < 0.05] and unimproved source of drinking water [AOR: 1.37; *p* < 0.05] were 22 per cent and 37 per cent more likely to suffer from water borne diseases respectively, compared to their counterparts. The likelihood of water borne diseases was 27 per cent and 16 per cent more among scheduled tribe [AOR: 1.27; *p* < 0.05] and other backward class elderly [AOR: 1.16; *p* < 0.05] respectively, compared to scheduled caste elderly. With reference to elderly who belonged to North region, the likelihood of water borne diseases was 36 per cent more among elderly who belonged to Central region [AOR: 1.36; *p* < 0.05].

**Table 4:** Logistic regression estimates for elderly who suffered from water borne diseases by their background characteristics in India

Background characteristics	AOR
	**95% CI**
**Place of residence**	
Rural	1.21*(1.12,1.31)
Urban	Ref
**Age (in years)**	
60–69	Ref
70–79	0.99(0.92,1.06)
80 +	0.94(0.85,1.05)
**Sex**	
Male	Ref
Female	1.19*(1.1,1.28)
**Education**	
No education/primary not completed	1.56*(1.34,1.82)
Primary completed	1.37*(1.16,1.62)
Secondary completed	1.31*(1.12,1.54)
Higher and above	Ref
**Marital status**	
Currently married	Ref
Widowed	1.05(0.98,1.13)
Others	0.92(0.75,1.13)
**Working status**	
Working	Ref
Retired	0.95(0.88,1.03)
Not working	0.81*(0.73,0.88)
**Body Mass Index**	
Underweight	1.12*(1.01,1.24)
Normal	1.08(0.99,1.17)
Overweight/obese	Ref
**Type of toilet facility**	
Unimproved	1.22*(1.13,1.32)
Improved	Ref
**Source of drinking water**	
Unimproved	1.37*(1.2,1.57)
Improved	Ref
**Type of house**	
Pucca	Ref
Semi pucca	1.14*(1.06,1.23)
Kutcha	1.08(0.99,1.19)
**MPCE quintile**	
Poorest	0.87*(0.78,0.96)
Poorer	1.02(0.93,1.13)
Middle	0.93(0.84,1.03)
Richer	1.01(0.91,1.11)
Richest	Ref
**Religion**	
Hindu	Ref
Muslim	0.92(0.83,1.01)
Christian	0.99(0.86,1.15)
Others	0.91(0.79,1.04)
**Caste**	
Scheduled Caste	Ref
Scheduled Tribe	1.27*(1.13,1.43)
Other Backward Class	1.16*(1.06,1.26)
Others	0.93(0.84,1.02)
**Region**	
North	Ref
Central	1.36*(1.23,1.5)
East	0.69*(0.63,0.76)
Northeast	0.46*(0.4,0.53)
West	0.50*(0.45,0.56)
South	0.23*(0.21,0.26)
**Cox & snell R square**	0.065
**Nagelkerke R square**	0.097

*Ref* Reference, ^*^ if *p* < 0.05, *CI* Confidence interval, *AOR* Adjusted Odds Ratio

## Discussion

The present study tries to see the prevalence and predictors of water borne disease in India. The prevalence of water borne disease among the elderly is more in the rural (22.5%) areas compared to the urban counterparts (12.2%) with a significant absolute difference of about 10.2%. The percentage of elderly population with waterborne disease is more in the central Indian states like Chhattisgarh and Madhya Pradesh followed by the North Indian states. The result of logistic regression concludes that sex of the participant, educational status, working status, BMI, place of residence, type of toilet facility and water source are important determinants of water borne disease among elderly in India. The infectious disease distribution which includes water borne diseases involves complex social and demographic factors including human population density and behaviour, housing type and location, water supply, sewage and waste management systems, land use and irrigation systems, access to health care, and general environmental hygiene [30]. In the study the waterborne diseases include diarrhoea, typhoid and jaundice. Earlier studies have shown diarrhoea and its complication to be more among elderly people, particularly those who require long term care [31]. The study finding that waterborne diseases are more in the rural areas compared to the urban areas is also consistent with earlier studies which concluded diarrheal prevalence to be more in rural areas and also in Central part of the country [32, 33]. A meta-analysis of typhoid prevalence in India concluded that this waterborne disease prevalence was more in the rural area with 0.09 lesser odds of having the disease in urban counterparts [34].

Our finding that waterborne disease prevalence vary with the anthropometric status as measured by BMI level with significantly higher odds of prevalence among the underweight compared to the overweight participants have theoretical justification as well. The relationship between malnutrition and the infection risk is bidirectional where infection adversely affects nutritional status through reductions in dietary intake and intestinal absorption, increased catabolism and sequestration of nutrients that are required for tissue synthesis and growth. On the other hand, malnutrition can predispose to infection because of its negative impact on the barrier protection afforded by the skin and mucous membranes and by inducing alterations in host immune function [35–37]. Earlier studies based on infectious disease risk among the children have indicated the educational status of mother as an important determinant with more infections among illiterate mothers [32, 38, 39]. The studies have debated that the disease risk is lesser among educated mothers because of hygiene practices, child feeding and caring practices, and improved living conditions. Similarly among the elderly participants as well educated people have a better understanding of the hygiene practices and feeding and caring habits and hence a reduced risk of waterborne infections.

The study finding that disease risk is more among population using unimproved sources of water and sanitation is consistent with earlier study which states drinking water, sanitation facilities and hygienic behaviour are the determining factors of health of household members [40]. A longitudinal study in the slums of Ethiopia shows sanitation facilities and hygienic condition of households were associated with acute diarrhoea [41]. Studies have also indicated improved water, sanitation and hygiene conditions of the households are accountable for diarrheal and other waterborne diseases [42, 43]. Unimproved sources of drinking water, quality of drinking water, absences of sanitation facilities and garbage collection was associated with stomach problem in urban India [16, 44, 45].

India suffers from a higher burden of infectious disease particularly water bone disease due to a weak public drinking water distribution system [10]. The degraded water quality can contribute to water scarcity as it limits its availability for both human use and for the ecosystem. With more than 8% of elderly aged 60 years and above residing in India [19] it is important to see the prevalence of water borne disease among the increasing population of elderly as there is a need to protect the population since treatment cost is also not cheap. Moreover, studies indicating infectious disease among the elderly is very few [21]. Thus the present analysis is an important contribution in research related to health of the elder population.

## Conclusion

Elderly living in rural areas are more prone to waterborne diseases. Use of unimproved water and absence of improved sanitation are major factors affecting waterborne disease among elderly. However the major limitation of the study is that the disease prevalence is based on self-reported morbidity status and lacks clinical verification, with a possibility of under reporting as well as over reporting and thus an underestimation or overestimation of the prevalence of the morbidities under study. However as is seen in various studies these self-reported measures or patient reported outcomes address issues that are of primary interest to the clinician and thus can be considered for measurement [23]. Consistent with findings from earlier literature regardless of whether there is under-reporting or over-reporting, the aforesaid socio-economic and demographic factors affect the pattern of morbidities associated with infections among elderly in India.

The elderly population might not be aware of the hygiene practices which adhere to the disease risk among this group. With age the antibody resistance falls and thus they might be well affected by the waterborne diseases. There is a need to focus on this population on preventing such bacterial diseases. This can be achieved by encouraging those aged 60 years and above as well as their caretakers to seek healthcare at early signs of infection. It also recommends making elderly aware of how to maintain the proper hygienic condition while availing the improved sanitation and water facilities provided to the people. The government should focus on providing safe water to the elderly population, train them to store water in a right and proper way.

## Supplementary Information

**Additional file 1.**

## Data Availability

https://www.iipsindia.ac.in/content/LASI-data
