# Supplementary material for: Prevalence and predictors of water-borne diseases among elderly people in India: evidence from Longitudinal Ageing Study in India, 2017–18
Source: BMC Public Health. 2022 May 17;22:993. doi: 10.1186/s12889-022-13376-6 (PMC9112585; doi:10.1186/s12889-022-13376-6)
Supplement: Supplementary file 1 — Additional file 1. [file 12889_2022_13376_MOESM1_ESM.pdf]

Table-A1. VIF score to check multicollinearity.

Variables	VIF
Age (in years)	1.12
Sex	1.54
Education	1.43
Marital status	1.21
Working status	1.39
Body Mass Index	1.07
Type of toilet facility	1.24
Source of drinking water	1.03
Type of House	1.33
MPCE quintile	1.16
Religion	1.08
Caste	1.16
Place of residence	1.34
Region	1.06

VIF: Variance Inflation Factor

Table-A2 Percentage of older adults suffered from water borne diseases in states of India (rural and urban)

States	Rural (%)	Urban (%)	p-value
Jammu & Kashmir	10.4	6.6	0.001
Himachal Pradesh	28.7	32.9	0.001
Punjab	22.0	23.9	0.108
Chandigarh	0.0	11.5	0.001
Uttarakhand	11.6	14.2	0.001
Haryana	35.2	32.4	0.001
Delhi	0.0	17.5	0.001
Rajasthan	34.9	25.3	0.001
Uttar Pradesh	33.6	26.7	0.001
Bihar	33.7	36.3	0.001
Arunachal Pradesh	24.6	9.9	0.001
Nagaland	1.2	2.8	0.001
Manipur	19.7	24.1	0.001
Mizoram	34.5	36.1	0.001
Tripura	13.5	8.6	0.001
Meghalaya	9.5	5.5	0.001
Assam	12.6	7.2	0.001
West Bengal	14.9	10.1	0.001
Jharkhand	18.1	12.5	0.001
Odisha	11.1	8.1	0.001
Chhattisgarh	38.5	30.4	0.001
Madhya Pradesh	36.0	26.1	0.001
Gujarat	22.3	14.5	0.001
Daman & Diu	20.1	12.8	0.001
Dadra & Nagar Haveli	44.9	42.0	0.001
Maharashtra	12.8	4.9	0.001
Andhra Pradesh	9.4	3.3	0.001

Karnataka	14.5	1.9	0.001
Goa	8.6	4.1	0.001
Lakshadweep	7.3	1.5	0.001
Kerala	3.9	3.5	0.665
Tamil Nadu	7.7	5.0	0.567
Puducherry	8.9	1.7	0.001
Andaman & Nicobar Island	26.0	18.2	0.001
Telangana	8.8	8.3	0.287
**India**	**22.5**	**12.2**	0.001
